# Supplementary material for: Super-Ballistic Width Dependence of Thermal Conductivity in Graphite Nanoribbons and Microribbons
Source: Nanomaterials (Basel). 2023 Jun 13;13(12):1854. doi: 10.3390/nano13121854 (PMC10301785; doi:10.3390/nano13121854)
Supplement: Supplementary file 1 [file nanomaterials-13-01854-s001.zip › nanomaterials-2429127-supplementary.pdf]

Supplementary Materials

Super-Ballistic Width Dependence of Thermal Conductivity in Graphite Nanoribbons and Microribbons

Xin Huang [1,*], Satoru Masubuchi [1], Kenji Watanabe [2], Takashi Taniguchi [1,3], Tomoki Machida [1] and Masahiro Nomura [1,*]

[1] Institute of Industrial Science, The University of Tokyo, Tokyo 153-8505, Japan
[2] Research Center for Electronic and Optical Materials, National Institute for Materials Science, 1-1 Namiki, Tsukuba 305-0044, Japan
[3] Research Center for Materials Nanoarchitectonics, National Institute for Materials Science, 1-1 Namiki, Tsukuba 305-0044, Japan
* Correspondence: huangxin@iis.u-tokyo.ac.jp (X.H.); nomura@iis.u-tokyo.ac.jp (M.N.)

Characterization of the roughness and width of the structures

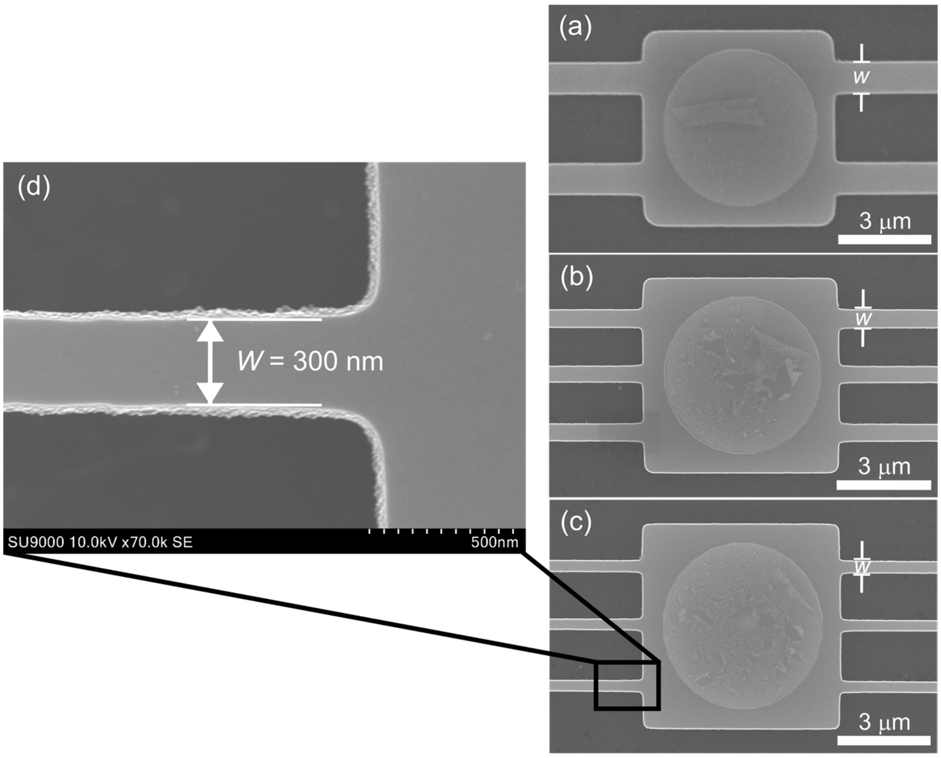

Figure S1. Scanning electron microscope (SEM) image of suspended graphite ribbons with the width (W) of (a) 1.2 mm, (b) 600 nm, and (c-d) 300 nm.
